# Inhibition of Selenoprotein I promotes ferroptosis and reverses resistance to platinum chemotherapy by impairing Akt phosphorylation in ovarian cancer

**DOI:** 10.1002/mco2.70033

**Published:** 2024-12-11

**Authors:** Jing Li, Mimi Chen, Dingwen Huang, Ziyin Li, Yu Chen, Jinhua Huang, Yuanqun Chen, Zhili Zhou, Zhiying Yu

**Affiliations:** ^1^ Department of Gynecology Shenzhen Second People's Hospital the First Affiliated Hospital of Shenzhen University Shenzhen China; ^2^ Guangdong Key Laboratory for Biomedical Measurements and Ultrasound Imaging National‐Regional Key Technology Engineering Laboratory for Medical Ultrasound School of Biomedical Engineering Shenzhen University Medical School Shenzhen China; ^3^ Department of Endocrinology and Metabolism Nanfang Hospital Southern Medical University Guangzhou China; ^4^ Shenzhen Key Laboratory of Reproductive Immunology for Peri‐implantation Shenzhen Zhongshan Institute for Reproductive Medicine and Genetics Shenzhen Zhongshan Obstetrics & Gynecology Hospital Shenzhen China

**Keywords:** Akt phosphorylation, chemoresistance, ferroptosis, ovarian cancer, SELENOI

## Abstract

Ovarian cancer (OV) ranks among the deadliest gynecological cancer, known for its high risk of relapse and metastasis, and a general resistance to conventional platinum‐based chemotherapy. Selenoprotein I (SELENOI) is a crucial mediator implicated in human hereditary spastic paraplegia. However, its role in human tumors remains poorly elucidated. Here, we comprehensively analyzed SELENOI expression patterns, functions, and clinical implications across various malignancies through the integration of bulk transcriptomics, cancer databases, and in vitro and in vivo experiments. Pan‐cancer analysis indicated upregulated SELENOI expression across various cancers, correlating with augmented malignancy, suppressed tumor immunity and poor prognosis. Knockdown of *SELENOI* caused G0/G1‐phase cell cycle arrest and diminished aggressive cancer phenotypes in OV cells. Moreover, SELENOI inhibition augments ferroptosis and reverses the cisplatin resistance in OV cells by modulating Akt phosphorylation. Conversely, overexpression of *SELENOI* in OV cells enhanced therapeutic sensitivity to cisplatin by upregulating Akt phosphorylation. Importantly, in vivo studies demonstrated that SELENOI inhibition suppressed ovarian tumor growth and enhanced cisplatin's anticancer effects. These findings highlight the significant role of SELENOI in OV by modulating ferroptosis and chemotherapy resistance. Targeting SELENOI represents a promising therapeutic approach to promote the efficacy of platinum‐based chemotherapy in OV, particularly in cases of resistance.

## Graphical abstract

Graphic summary of targeting SELENOI for promoting ferroptosis and reversing chemoresistance in ovarian cancer. Inhibition of SELENOI activated unsaturated fatty acid synthesis and impaired Akt phosphorylation, resulting in the upregulation of ACSL4 expression and induction of ferroptosis. Reduced Akt phosphorylation also led to the downregulation of ALDH3A1 expression, a key gene associated with cisplatin‐resistance, thereby enhancing cisplatin cytotoxicity in SELENOI‐silenced ovarian cancer cells.

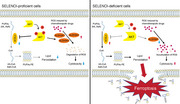

## 1 INTRODUCTION

Ovarian cancer (OV), the most lethal gynecological malignancy, is characterized by advanced‐stage diagnosis, frequent recurrence, chemotherapy resistance, and poor outcomes.^1^, ^2^, ^3^ The incidence and mortality rates of OV are rising globally, particularly among younger women.^4^ Paclitaxel combined with platinum‐based chemotherapy remains the preferred postcytoreduction strategy.^3^, ^5^ However, over 80% of patients experience relapse and develop platinum resistance, particularly in advanced stages.^6^, ^7^ While PARP inhibitors, angiogenesis inhibitors, and immunotherapy are increasingly used, not all patients respond to these therapies.^8^, ^9^ Effective strategies for managing OV recurrence and metastasis remain limited, highlighting the urgent need for novel therapeutic targets and interventions.

Selenium, an essential trace element, contributes to maintaining redox homeostasis through selenoproteins.^10^, ^11^ Among them, glutathione peroxidase 4 (GPX4) is crucial in regulating ferroptosis by converting lipid peroxides into nontoxic lipid alcohols and abrogating ferroptotic cell death.^12^ Selenium also reduces mitochondrial ubiquinone to ubiquinol preventing lipid peroxidation and ferroptosis.^13^ This highlights selenium and selenoproteins’ critical roles in antioxidant defense and ferroptosis regulation. Inducing ferroptosis has shown significant antitumor effects in various cancers and may enhance the efficacy of traditional chemotherapeutics and overcome chemoresistance.^14^, ^15^, ^16^, ^17^ As a key event in ferroptosis, lipid peroxidation preferentially oxidizes polyunsaturated fatty acids (PUFAs)‐rich phosphatidylethanolamine (PE).^12^, ^18^ Acyl‐CoA synthetase long‐chain family member 4 (ACSL4) is essential in this process, incorporating free PUFAs—primarily arachidonic acid and adrenic acid—into membranous PE.^19^ Selenoprotein I (SELENOI), a member of the CDP‐ethanolamine phosphatidyltransferase class I family, is involved in de novo phospholipid synthesis through the Kennedy pathway,^20^ catalyzing the final step that generates various PE species.^21^, ^22^ Recent studies have identified SELENOI's crucial role in maintaining ether‐linked PE (plasmalogens) homeostasis and promoting nerve myelination.^23^, ^24^ Dysfunctions in SELENOI are linked to hypomyelination, ether‐linked PE deficiency,^23^ and hereditary spastic paraplegia, a severe neurogenetic disorder.^24^ CD4^+^ T cell differentiation also relies on key enzymes of the Kennedy pathway, including ETNK1, PCYT2, and SELENOI.^25^, ^26^ While SELENOI is crucial for lipid metabolism and neurological function, its role in human cancer remains unclear.

In this study, we identified *SELENOI* as a key regulator of ferroptosis, demonstrating its upregulation in multiple human cancers. Elevated *SELENOI* expression correlates with increased tumor malignancy, advanced stages and poorer prognosis, potentially impacting the response to immunotherapy. *SELENOI* knockdown reduced cell viability and suppressed aggressive cancer phenotypes in OV cells. Furthermore, silencing SELENOI enhanced OV cells’ sensitivity to ferroptosis agonists and reversed cisplatin resistance by modulating Akt phosphorylation and its downstream targets. These findings highlight SELENOI as a critical driver of tumor progression and a promising target in OV therapy.

## 2 RESULTS

### 2.1 Identification of SELENOI as a potential regulator of ferroptosis in OV through comprehensive transcriptome analysis

**FIGURE 1:**
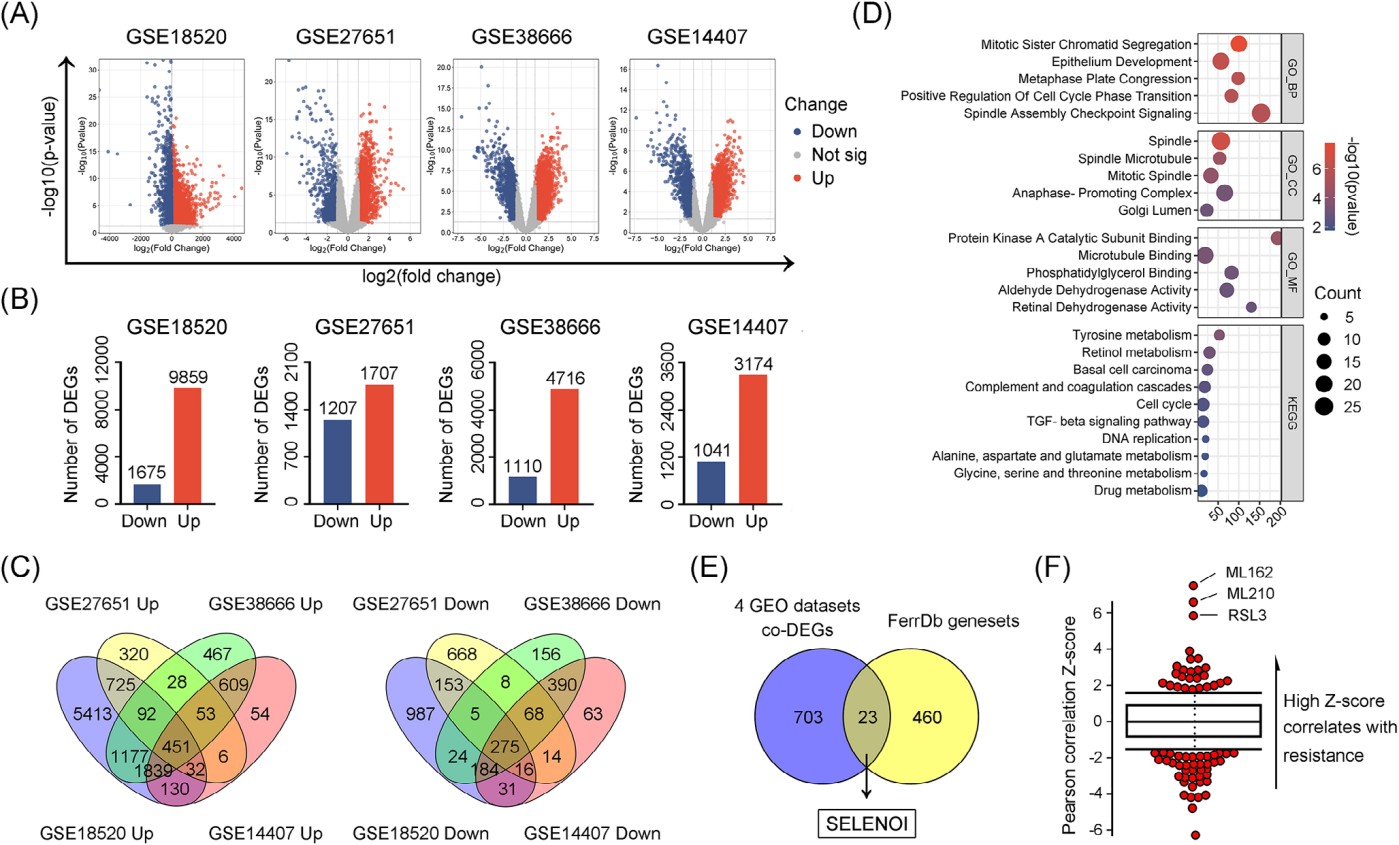
Identification of *SELENOI* as a potential ferroptosis regulatory gene in OV. (A and B) Volcano plots and histograms illustrating the shared DEGs in the four OV datasets. (C) Venn diagram showing overlapping DEGs between the four OV datasets. (D) GO and KEGG enrichment analyses of overlapping DEGs. (E) Venn diagram showing the overlapping genes between 726 shared DEGs identified in four OV datasets and 483 genes in the ferroptosis dataset (FerrDb). (F) CTRP database revealed the highest correlation between *SELENOI* expression and the resistances of three ferroptosis inducers (RSL3, ML162, and ML210).

Given the high drug resistance and lethality of OV, we conducted a limma differential gene expression analysis across four OV datasets to identify common therapeutic targets (Figure 1A,B). We identified 451 shared upregulated and 275 downregulated differentially expressed genes (DEGs) based on |log_2_foldchange| > 1 and *p* value < 0.05 criteria (Figure 1C and Table S1). Gene Ontology (GO) and Kyoto Encyclopedia of Genes and Genomes (KEGG) enrichment analyses revealed that these DEGs were primarily involved in mitosis, cell cycle regulation, and amino acid and retinol metabolism pathways (Figure 1D).

Inducing ferroptosis has emerged as a novel strategy to overcome chemotherapy resistance in solid tumors.^17^ To identify ferroptosis‐related DEGs in OV cells, we interrogated the FerrDb ferroptosis dataset and performed an overlapping analysis with 726 DEGs identified. A total of 23 genes potentially involved in ferroptosis regulation were identified (Figure 1E). Among them, *SELENOI*, a crucial gene in PE synthesis via the Kennedy pathway, showed the strongest correlation with resistance to three ferroptosis inducers (ML210, ML162, and RSL3) based on CTRP drug response data (Figure 1F). Given that lipid peroxidation, particularly of PUFAs‐rich PE species, is central to ferroptosis, *SELENOI* was identified as a potential ferroptotic gene.

### 2.2 SELENOI was significantly upregulated in female malignancies and predicted poorer survival

**FIGURE 2:**
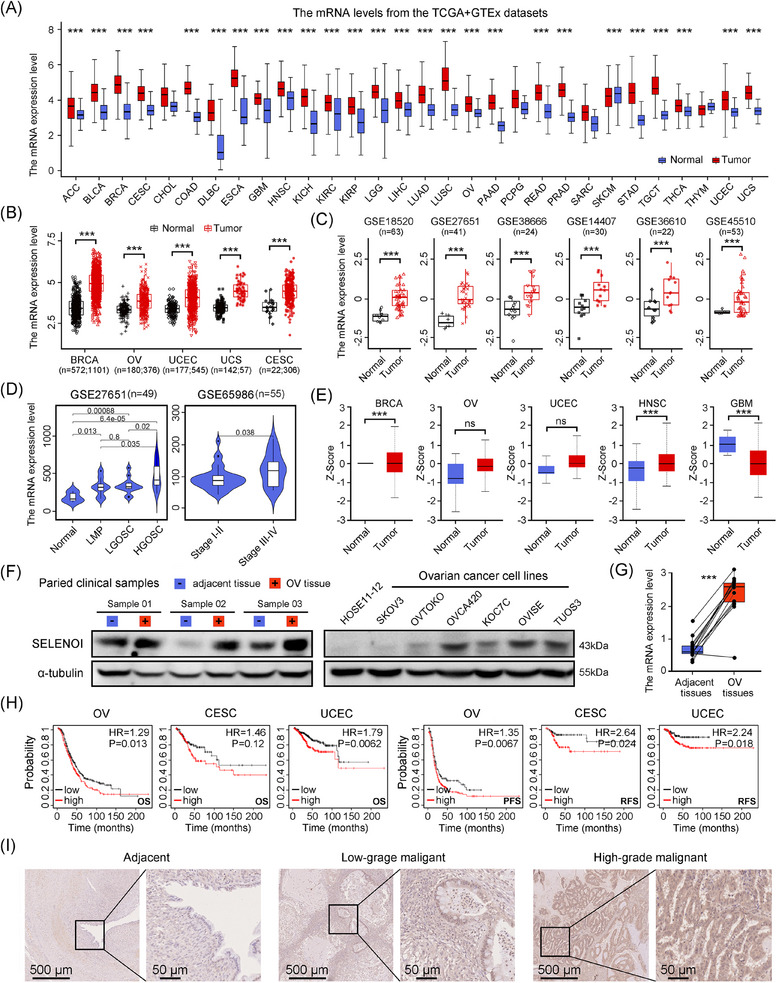
*SELENOI* expression was upregulated in various cancers and correlated with adverse clinical outcomes. (A) Analysis of *SELENOI* mRNA expression in various cancers from the TCGA and GTEx databases (unpaired samples). (B and C) *SELENOI* mRNA expression levels between tumors and normal tissues in five cancer types from TCGA database and in two cancer types from GEO database. (D) Correlation between *SELENOI* mRNA expression and tumor malignancy and stage in two OV datasets from GEO database. (E) Analysis of SELENOI protein expression between normal and primary tumor tissues on UALCAN. (F and G) Western blotting and qPCR analysis of *SELENOI* expression in paired normal and cancer tissues from our OV cohort (*n* = 30) and a set of cancer cell lines. α‐tubulin was used as the loading control. (H) Survival analysis of cancer patients with high and low *SELENOI* expression using the Kaplan–Meier plotter tool. OS, overall survival; PFS, progression‐free survival; RFS, recurrence‐free survival. (I) SELENOI immunohistochemical staining (IHC) of adjacent normal tissues, low‐grade and high‐grade malignant tumor tissue samples from ovarian cancer patients. ^*^ *p* < 0.05, ^**^ *p* < 0.01, ^***^ *p* < 0.001. ACC, adrenocortical carcinoma; BLCA, bladder urothelial carcinoma; BRCA, breast invasive carcinoma; CESC, cervical squamous cell carcinoma and endocervical adenocarcinoma; CHOL, cholangio carcinoma; COAD, colon adenocarcinoma; DLBC, lymphoid neoplasm diffuse large B‐cell lymphoma; ESCA, esophageal carcinoma; GBM, glioblastoma multiforme; HNSC, head and neck squamous cell carcinoma; KICH, kidney chromophobe; KIRC, kidney renal clear cell carcinoma; KIRP, kidney renal papillary cell carcinoma; LAML, acute myeloid leukemia; LGG, brain lower grade glioma; LIHC, liver hepatocellular carcinoma; LUAD, lung adenocarcinoma; LUSC, lung squamous cell carcinoma; MESO, mesothelioma; OV, ovarian carcinoma; PAAD, pancreatic adenocarcinoma; PCPG, pheochromocytoma and paraganglioma; PRAD, prostate adenocarcinoma; READ, rectum adenocarcinoma; SARC, sarcoma; SKCM, skin cutaneous melanoma; STAD, stomach adenocarcinoma; TGCT, testicular germ cell tumors; THCA, thyroid carcinoma; THYM, thymoma; UCEC, uterine corpus endometrial carcinoma; UCS, uterine carcinosarcoma; UVM; uveal melanoma.

Given the limited understanding of SELENOI's role in cancer, we assessed its expression in human malignancies using the Cancer Genome Atlas (TCGA) and the Genotype‐Tissue Expression (GTEx) databases. *SELENOI* expression was significantly elevated in nearly all cancers (Figure 2C and Table S2). Compared with adjacent non‐neoplastic tissues, *SELENOI* mRNA levels were significantly upregulated in several female cancers (breast, ovarian, cervical, endometrial, and uterine sarcoma) (*p* < 0.001) (Figure 2D). Analysis of GEO database also revealed higher SELENOI expression in ovarian and uterine sarcoma tumors compared with normal tissues (Figure 2D), both of which are among the most lethal gynecological malignancies. When stratifying ovarian tumors by tumor properties, *SELENOI* expression increased with tumor malignancy (*p* < 0.05) (Figure 2E). The highest *SELENOI* mRNA levels were observed in high‐grade serous carcinomas, the most common histotype of OV. Additionally, higher *SELENOI* levels were observed in advanced‐stage (III and IV) tumors compared with earlier stages (I and II) (*p* < 0.05) (Figure 2E). Consistently, SELENOI protein expression was elevated across various cancers, particularly in female tumors (BRCA, OV, and UCEC) (Figure 2F). Upregulated *SELENOI* transcript and protein levels were also confirmed in tumor tissues from our OV cohort and cancer cell lines (Figure 2G,H). Higher SELENOI levels were significantly associated with reduced overall survival and shorter progression‐free survival in three gynecological malignancies (Figure 2I). Additionally, immunohistochemical analysis further validated increased SELENOI expression in OV tissues compared with adjacent nontumorous tissues (Figure 2I). These findings suggest SELENOI may play a significant role in the progression of female malignancies, particularly OV.

### 2.3 SELENOI expression was associated with tumor immune checkpoint‐related genes, tumor immune cell infiltration, and the tumor microenvironment

**FIGURE 3:**
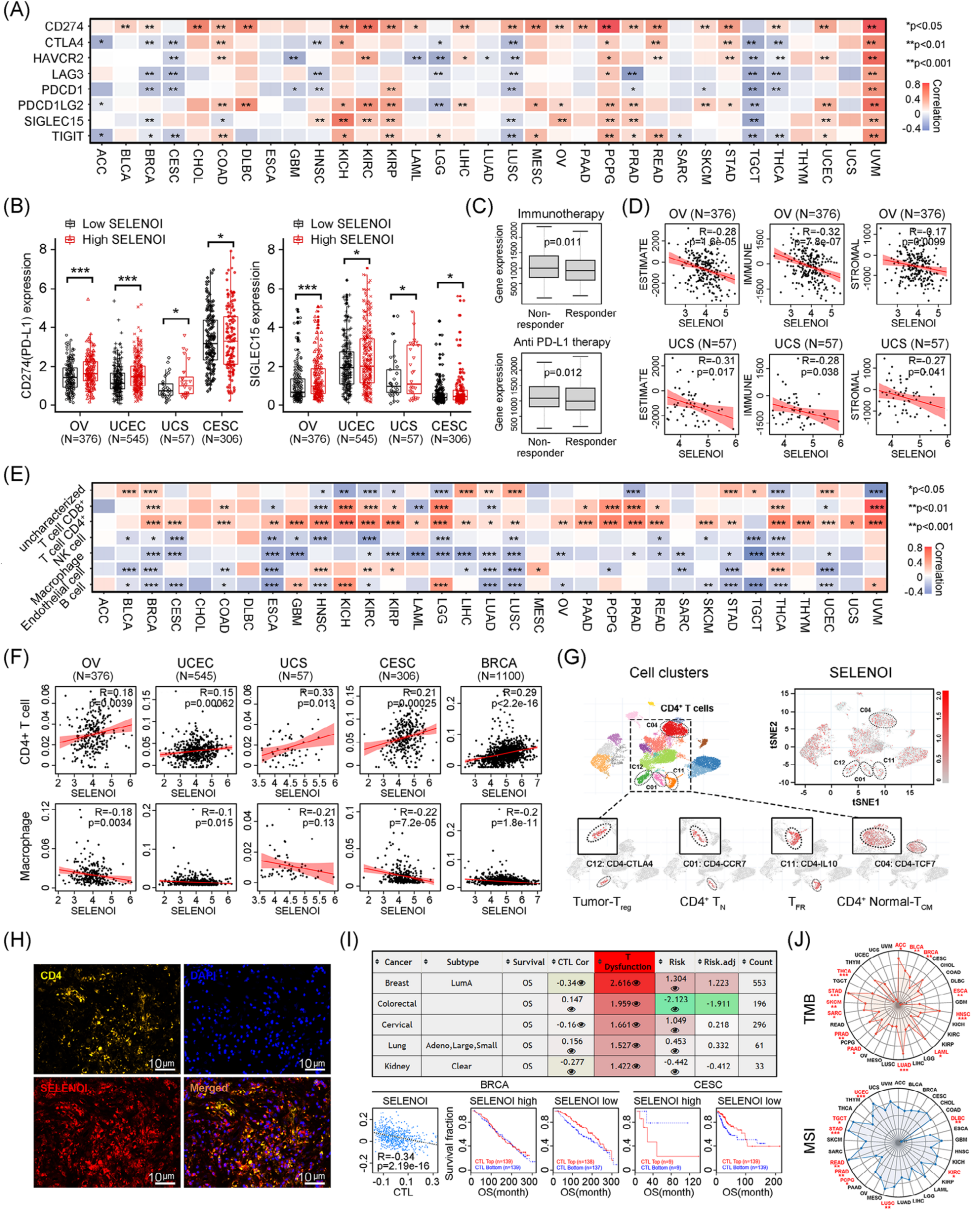
SELENOI expression was related to tumor immunity in pan‐cancer. (A) Heat map showing the correlation between *SELENOI* and key immune checkpoint genes’ expression in pan‐cancer. (B) Histogram showing the difference of *CD274* and *SIGLEC15* expression between *SELENOI*‐high and ‐low groups in four gynecological tumors. (C) Differences of *SELENOI* expression between immunotherapy responders and nonresponders in OV using ROCplotter. (D) Correlation between *SELENOI* expression and estimate score, immune score, and stromal score in OV and UCS using ESTIMATE. (E) Heat map showing the correlation between *SELENOI* expression and the infiltration levels of six immune cells based on the EPIC algorithm. (F) Correlation coefficient scatter plot showing *SELENOI* expression and CD4^+^ T cell and macrophage infiltration levels in five female tumors. (G) Analysis of *SELENOI* expression and intrinsic heterogeneity of CD4^+^ T cells based on the single‐cell dataset GSE181297 using CDCP. Each point corresponds to a cell and is colored according to cell clusters. Color density indicates the expression level of *SELENOI*. (H) Double immunofluorescence staining of SELENOI (red) and CD4 (yellow) in OV tissues. DAPI staining is shown in blue. Scale bar: 10 µm. (I) Analysis of correlations between *SELENOI* expression and the cytotoxic T lymphocytes, T cell dysfunction, and risk using TIDE. (J) Radar plot representing the correlation between *SELENOI* expression and TMB and MSI in pan‐cancer using R maftools. ^*^ *p* < 0.05, ^**^ *p* < 0.01, ^***^ *p* < 0.001.

Immune cells enriched in tumor microenvironment, including macrophages and T cells, facilitate ferroptosis by producing reactive oxygen species (ROS) or secreting IFN‐γ.^27^ Recent research has identified key enzymes involved in CD4^+^ T cell differentiation and the Kennedy pathway—ETNK1, PCYT2, and SELENOI—suggesting a potential correlation between SELENOI and tumor immunity.^25^, ^26^ Therefore, we investigated the impact of SELENOI on tumor immune responses. *SELENOI* expression positively correlated with immune checkpoint genes in UVM, PCPG, and KICH, but negatively correlated in TGCT, BRCA, and LUSC (*p* < 0.05) (Figure 3A). Notably, high SELENOI expression was associated with upregulated *CD274* (PD‐L1) and *SIGLEC15* in gynecological malignancies(*p* < 0.05) (Figure 3B), which are mutually exclusive and suppress effector T cells through distinct mechanisms.^28^ In OV patients, high *SELENOI* expression were associated with nonresponsiveness to immunotherapy, including anti‐PD‐L1 therapy (*p* < 0.05) (Figure 3C). Low *SELENOI* expression in OV and UCS was related to increased stromal and immune cells’ abundance, as indicated by significantly higher ESTIMATE scores (Figure 3D).

Additionally, *SELENOI* expression was positively correlated with CD4^+^ T cells and negatively correlated with macrophages in most cancers (*p* < 0.05) (Figure 3E,F). Single‐cell analysis (GSE181297) confirmed higher *SELENOI* expression in several CD4^+^ T cell clusters (Figure 3G), suggesting a role in supporting CD4^+^ T cell function. Double immunofluorescence staining of OV tissues revealed that CD4^+^ T cell (yellow) accumulated in areas of high SELENOI expression (red) (Figure 3H). Cytotoxic T lymphocytes (CTLs), the primary cells affected during immunosuppression, was also linked to *SELENOI* expression. Negative correlations between CTLs and SELENOI were observed in CESC, BRCA, and KIRC (Figure 3I). We further explored the relationship between *SELENOI* expression, TMB, and MSI, as these genomic alterations influence clinical outcomes and treatment response.^29^ SELENOI was positively correlated with TMB in multiple cancers, including LUAD, STAD, HNSC, and BRCA (Figure 3J). Regarding MSI, *SELENOI* showed positive correlations in UCEC, READ, LUSC, TGCT, KIRC, and STAD, while negative correlations were observed in DLBC, PRAD, and PCPG (Figure 3J). These findings suggest that SELENOI could serve as a potential biomarker for predicting immunotherapy responses, and inhibiting SELENOI might enhance the efficacy of antitumor immunotherapies.

### 2.4 SELENOI was involved in pathways related to fatty acid and phospholipid metabolism, as well as the cell cycle

**FIGURE 4:**
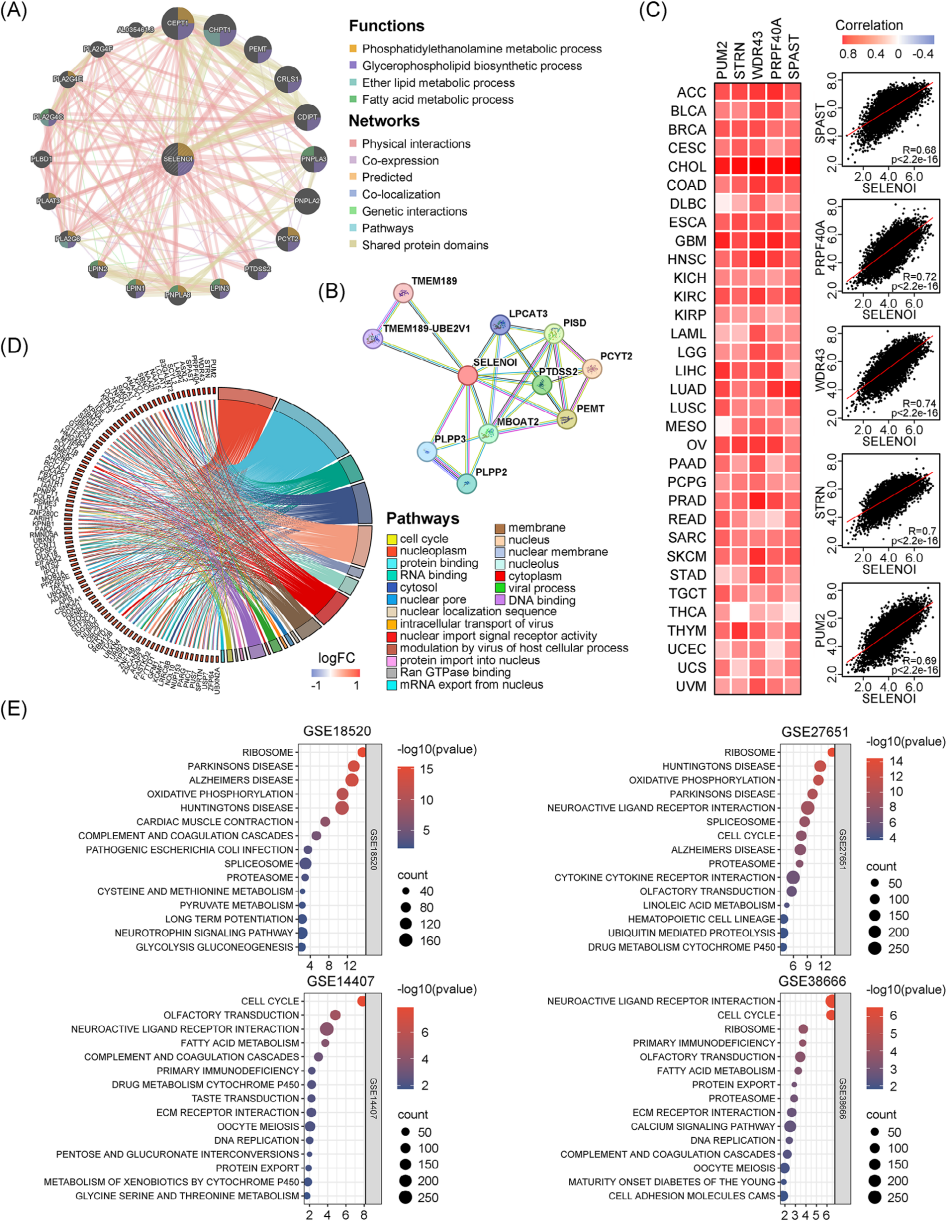
SELENOI was involved in fatty acid, phospholipid metabolism and cell cycle related pathways. (A) Analysis of *SELENOI*’s GGI network and its functions using GeneMANIA. (B) Analysis of SELENOI's PPI network and 10 significantly related proteins using STRING. (C) Heat map (left) and scatter plot (right) showing the correlation between *SELENOI* and the top 5 SELENOI coexpressed genes identified in pan‐cancer using GEPIA2.0. (D) Circle plots showing the enriched GO terms of top 100 *SELENOI* coexpressed genes. (E) The enriched KEGG pathways in four OV datasets from the GEO database using GSEA. Grouping based on median expression of *SELENOI*.

Given SELENOI's upregulation in solid tumors and its association with poor prognosis, we hypothesize that SELENOI may influence cell proliferation, migration, and cell cycle. Previous studies demonstrated the critical regulation of ferroptosis by fatty acid and phospholipid metabolism, where PUFAs are incorporated into the phospholipid membrane via PE and serve as crucial substrates for lipid peroxidation. Enzymes such as LPCAT3 and PI3K, regulate the cell's response to ferroptosis by modulating phospholipid metabolism and signaling.^30^, ^31^, ^32^ To elucidate SELENOI's interaction network and biological functions, we constructed a gene–gene interaction (GGI) network using GeneMANIA (Figure 4A). Twenty nodes surround the central *SELENOI* node, each representing a gene associated with *SELENOI* through shared protein domains, genetic interactions, physical interactions, colocalization, coexpression, or pathways. The top five genes associated with *SELENOI* are *CEPT1*, *CHPT1*, *PCYT2*, *CRLS1*, and *PEMT*. Functional analysis revealed that these genes predominantly participate in glycerophospholipid metabolism, including fatty acid metabolism, PE synthesis, and ether lipid synthesis.

We also constructed a protein–protein interaction (PPI) network using STRING (Figure 4B). Consistent with the GGI findings, PPI network also identified PCYT2, PEMT, and PISD as the top proteins interacting with SELENOI. Additionally, we analyzed the top 100 genes coexpressed with SELENOI across 33 cancer types using GEPIA2.0, and identified *PUM2*, *STRN*, *WDR43*, *PRPF40A*, and *SPAST* as highly correlated with *SELENOI* (Figure 4C). GO enrichment analysis showed that these coexpressed genes are involved in nuclear and macromolecule binding transport (Figure 4D). Using Gene Set Enrichment Analysis (GSEA), we performed KEGG pathway enrichment analysis on four OV datasets, revealing significant associations between *SELENOI* expression and pathways related to the cell cycle, oxidative phosphorylation, and fatty acid metabolism (*p* < 0.05) (Figure 4E). These results suggest SELENOI's crucial role in regulating the cell cycle and metabolism of phospholipids and fatty acids in human tumors.

### 2.5 Knockdown of SELENOI attenuated the malignant phenotype of OV cells and arrested the cell cycle

**FIGURE 5:**
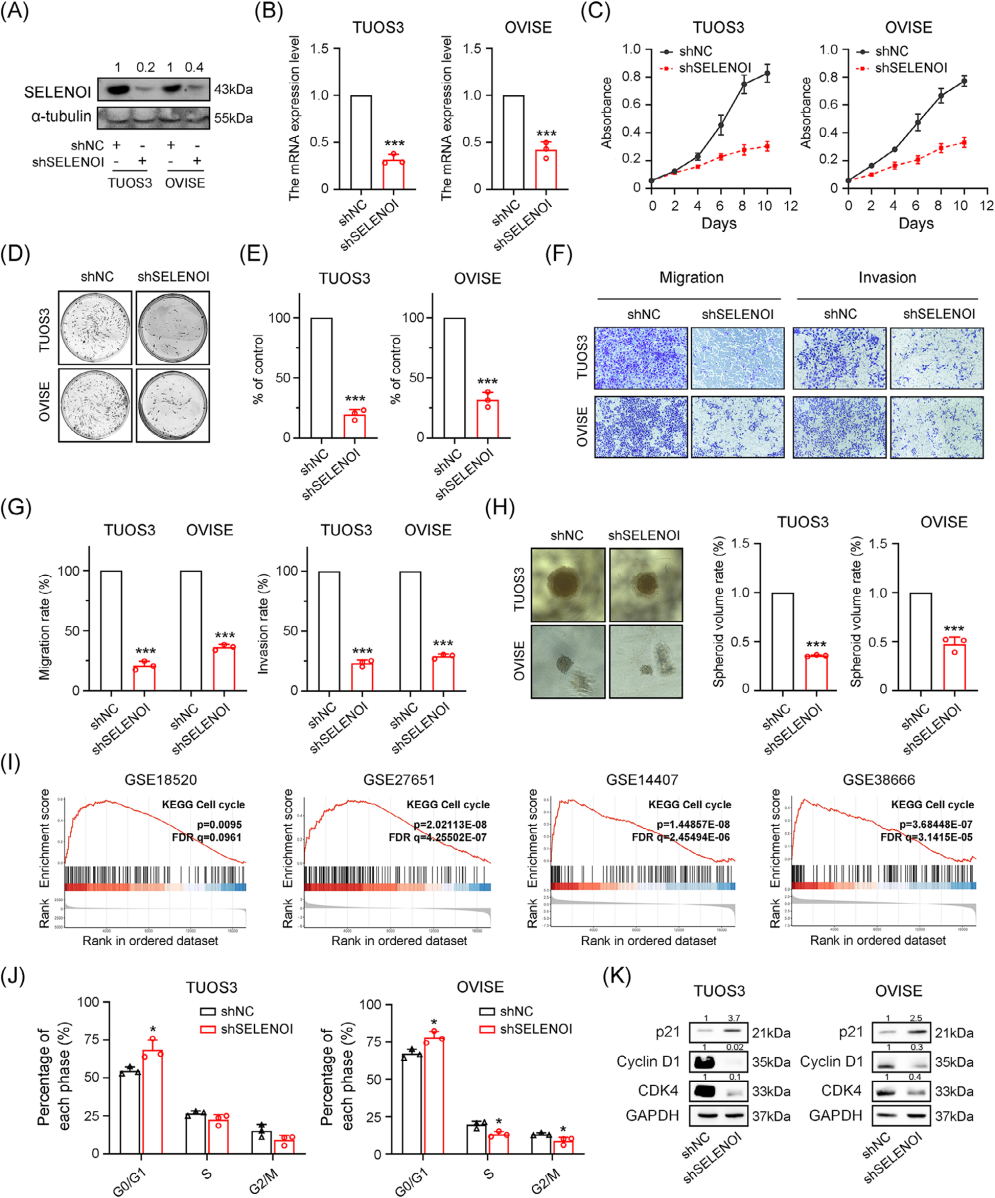
*SELENOI* knockdown attenuated the malignant phenotype formation of OV cells and promoted cell cycle arrest. (A and B) Western blotting and qPCR analysis confirmed that SELENOI protein and mRNA levels were significantly downregulated in *SELENOI* knockdown OV cells TUOS3 and OVISE. (C) Cell viability analysis of *SELENOI* knockdown (shSELENOI) and control OV (shNC) cells. (D and E) Colony formation analysis of shNC and shSELENOI OV cells. (F and G) Migration and invasion analysis of shNC and shSELENOI OV cells. (H) 3D sphere formation analysis of shNC and shSELENOI OV cells. (I) GSEA enrichment results of cell cycle signaling pathways in four OV datasets from the GEO database. Grouping based on median expression of *SELENOI*. (J) Changes in the G0/G1, S, and G2/M phases of the cell cycle in shNC and shSELENOI OV cells. (K) Western blotting of cyclin CDK4, P21, and Cyclin D1 levels in shNC and shSELENOI OV cells. GAPDH was used as loading control. ^*^ *p* < 0.05, ^**^ *p* < 0.01, ^***^ *p* < 0.001. Data represent means ± SD collected from three independent experiments.

Platinum‐based chemotherapy resistance is a major cause of therapeutic failure and poor outcomes in OV. Inducing ferroptosis presents a promising strategy to overcome chemoresistance in solid tumors. Given SELENOI's high expression and its association with bad prognosis, and proposed role in regulating ferroptosis and cell cycle, we investigated whether targeting SELENOI could influence the cell cycle, ferroptosis, and cisplatin responsiveness in OV cells. Human cisplatin‐resistant OV cells TUOS3 and OVISE,^33^ which naturally express high levels of *SELENOI*, were used to assess the effect of *SELENOI* knockdown on the transformed phenotype. SELENOI expression was inhibited using lentiviral plasmid constructs carrying commercially validated shRNAs targeting *SELENOI* (Figure 5A,B). Compared with control cells, *SELENOI* knockdown markedly inhibited cell viability, clonogenic survival, migration, invasion, as well as sphere‐forming ability (all *p* < 0.001) (Figure 5C–H). These results indicated that genetic silencing of *SELENOI* could attenuate aggressive cancer phenotypes in OV cells.

In addition, GSEA analysis of four OV RNA‐sequencing (RNA‐seq) datasets revealed a significant association between SELENOI expression and cell cycle (Figures 4E and 5I). We further evaluated the cell cycle distribution of OV cells and found that *SELENOI* knockdown increased the proportion of cells in the G0/G1‐phase (*p* < 0.05) (Figure 5J), with western blotting analysis showing increased p21 and decreased CDK4 and Cyclin D1 expression (Figure 5K). These findings suggest that *SELENOI* knockdown suppresses malignant phenotypes in OV cells and promotes cell cycle arrest.

### 2.6 Silencing of SELENOI promoted ferroptosis induced by erastin and sorafenib

**FIGURE 6:**
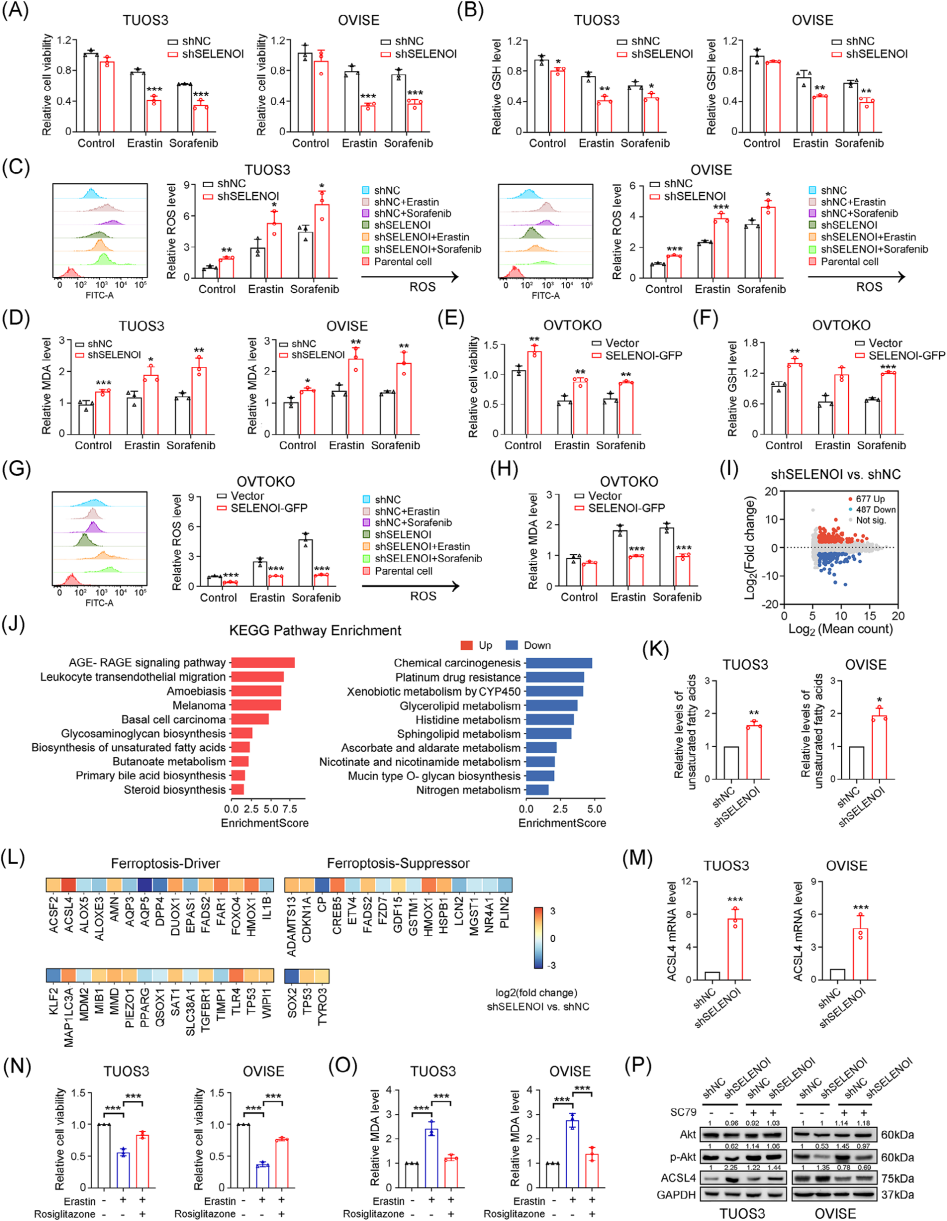
SELENOI regulated ferroptosis induced by erastin and sorafenib through upregulating the unsaturated fatty acids synthesis and ACSL4 expression in OV cells. (A–D) Analysis of the cell viability (A), GSH (B), ROS (C), and MDA (D) changes in shNC and shSELENOI TUOS3 and OVISE cells after 24 h treatment with erastin (10 µM) or sorafenib (10 µM) for 24 h. (E–H) Analysis of the cell viability (E), GSH (F), ROS (G) in *SELENOI* overexpressed (SELENOI‐GFP) and control (vector) OVTOKO cells after 24 h treatment with erastin (10 µM) or sorafenib (10 µM) and MDA (H) changes. (I) MA plot showing the DEGs identified between shNC and shSELENOI TUOS3 cells. (J) KEGG pathway enrichment analysis of significantly upregulated and downregulated signaling pathways caused by *SELENOI* knockdown. (K) Changes of total unsaturated fatty acids levels in *SELENOI* knockdown and control cells. (L) Heat map showing the expression changes of ferroptosis regulatory genes in *SELENOI* knockdown TUOS3 cells. (M) qPCR analysis confirmed that *ACSL4* mRNA levels were significantly upregulated in *SELENOI* knockdown TUOS3 and OVISE cells. (N and O) Cell viability and MDA changes in *SELENOI* knockdown cells treated with erastin and/or ACSL4 inhibitor rosiglitazone (10 µM) for 24 h. (P) Western blotting of Akt, p‐Akt, and ACSL4 expression levels in *SELENOI* knockdown and control cells treated with erastin and/or the Akt agonist SC79 (5 µM) for 24 h. GAPDH was used as loading control. ^*^ *p* < 0.05, ^**^ *p* < 0.01, ^***^ *p* < 0.001. Data represent means ± SD collected from three independent experiments.

To elucidate SELENOI's role in ferroptosis, we assessed cell death induced by erastin and sorafenib, two known ferroptosis inducers.^17^, ^34^ Knockdown of *SELENOI* in TUOS3 and OVISE cells significantly increased cell death caused by these agents (*p* < 0.001) (Figure 6A). We next investigated the role of SELENOI in regulating ferroptosis by analyzing ROS status and lipid peroxidation‐related metabolites. *SELENOI* inhibition markedly decreased glutathione (GSH) levels (Figure 6B) in these cells treated with sorafenib and erastin, while increasing ROS (Figure 6C) and lipid peroxidation metabolites malondialdehyde (MDA) levels (*p* < 0.05) (Figure 6D), which are two essential events in ferroptosis. Conversely, *SELENOI* overexpression in OVTOKO cells, which naturally express low endogenous *SELENOI* levels (Figures 2F and S2), significantly reduced erastin‐ and sorafenib‐induced cell death (*p* < 0.05) (Figure 6E), and decreased MDA and ROS accumulation while preserving GSH levels (Figure 6F–H). Transcriptomic sequencing analysis of *SELENOI* knockdown and control TUOS3 cells identified 677 significantly upregulated and 487 downregulated genes (Figure 6I) (Table S3). KEGG pathway analysis revealed that silencing of *SELENOI* activated the unsaturated fatty acid synthesis pathway, and inhibited the glycerophospholipid metabolism and platinum resistance pathways (Figure 6J). qPCR confirmed the upregulation of unsaturated fatty acids synthesis genes in *SELENOI*‐knockdown cells (Figure S2). Consistently, unsaturated fatty acids levels were also significantly elevated in these cells (Figure 6K). To further validate the ferroptosis‐specific candidates, we then cross‐referenced the 1164 DEGs with ferroptosis‐related genes from the FerrDb database. Although the expression levels of key ferroptosis regulators like GPX4 and SLC7A11 did not change significantly, *ACSL4*, a core driver of lipid peroxidation, was significantly upregulated in shSELENOI cells (Figure 6L,M). Notably, treatment with ACSL4 inhibitor rosiglitazone effectively reversed Erastin‐induced ferroptosis and reduced MDA levels in SELENOI‐silenced cells (Figure 6N,O). These results suggested that *SELENOI* knockdown promoted ferroptosis in OV cells via upregulating unsaturated fatty acids synthesis and ACSL4.

Hyperactive PI3K/Akt signaling confers resistance to oxidative stress and ferroptosis in cancer cells,^32^ Interestingly, *SELENOI* knockdown reduced phosphorylated Akt (p‐Akt) levels at ser473 without affecting total Akt in TUOS3 and OVISE cells (Figure 6P). *SELENOI* silencing also led to increased ACSL4 protein expression, while treatment with Akt agonist SC79 restored p‐Akt levels and suppressed ACSL4 expression (Figure 6P). Conversely, *SELENOI* overexpression in OVTOKO cells increased Akt phosphorylation and decreased ACSL4 levels compared with control cells (Figure S3). These findings suggest that *SELENOI* knockdown promotes ferroptosis in OV cells by upregulating ACSL4, potentially through Akt phosphorylation modulation.

### 2.7 Knockdown of SELENOI enhances the antitumor effect of cisplatin in OV cells

**FIGURE 7:**
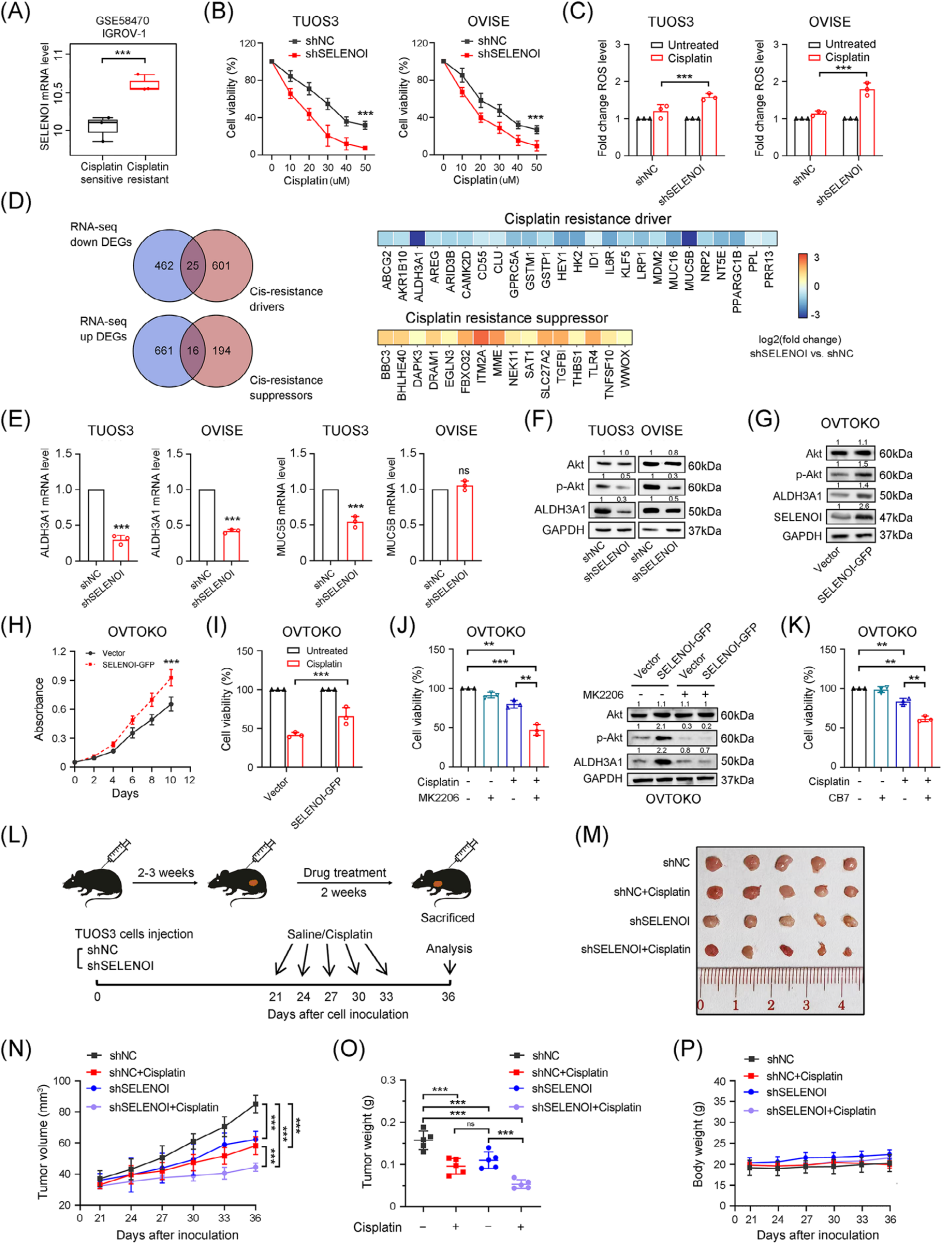
SELENOI regulated the antitumor effect of cisplatin in OV cells through inhibiting ALDH3A1 expression. (A) GSE58470 dataset analysis confirmed the upregulation of *SELENOI* expression in platinum‐resistant ovarian cancer cell line IGROV‐1 induced by cisplatin exposure compared with parental platinum‐sensitive cells. (B) Cell viability changes in shNC and shSELENOI OV cells after 10 µM cisplatin treatment for 24 h. (C) ROS analysis of shNC and shSELENOI OV cells after 10 µM cisplatin treatment for 24 h. (D) Venn diagram (left) showing the overlapping genes of shared DEGs in four OV datasets and the resistance‐related genes from the HESOC‐Platinum database. The heat map (right) showing the expression changes of the overlapping genes in shSELENOI TUOS3 cells compared with shNC cells. (E) qPCR analysis of *ALDH3A1* and *MUC5B* expression in shNC and shSELENOI cells. (F and G) Western blotting of Akt, p‐Akt, and ALDH3A1 expression levels in *SELENOI* knockdown, overexpression, and their control cells. (H and I) Cell viability analysis of *SELENOI* overexpression (SELENOI‐GFP) and control cells (vector) with/without 10 µM cisplatin treatment for 24 h. (J) Cell viability and western blotting analysis of Akt, p‐Akt, and ALDH3A1 expression levels in *SELENOI* overexpression and control cells treated with 10 µM cisplatin and/or 5 µM MK2206 for 24 h. GAPDH was used as loading control. (K) Cell viability analysis of *SELENOI* overexpression and control cells after treatment with single cisplatin (10 µM), or combined with ALDH3A1 inhibitor CB7 (5 µM) for 24 h. (L) Schematic representation of cisplatin treatment in vivo. (M and N) Changes in tumor volume (mm^3^) in each group of mice treated with cisplatin. (O) Changes in tumor weight (grams) of mice in each group after cisplatin treatment (5 mg/kg). (P) Time course plot showing changes in body weight (grams) for each group of mice treated with cisplatin. ^*^ *p* < 0.05, ^**^ *p* < 0.01, ^***^ *p* < 0.001; ns, not significant. Data represent means ± SD collected from three independent experiments.

Excessive activation of the PI3K/Akt pathway contributes to cisplatin resistance by preventing the propagation of DNA damage signals to the apoptotic mechinery.^35^, ^36^ In this study, *SELENOI* knockdown impaired Akt activation and caused G1 phase arrest, the most cisplatin‐sensitive stage. Transcriptomic analysis revealed that *SELENOI* knockdown downregulated the platinum resistance pathway in OV cells (Figure 6J). Additionally, molecular profiling of platinum‐sensitive and ‐resistant OV cells (GSE58470) showed that, *SELENOI* expression was significantly upregulated in cisplatin‐resistant IGROV‐1/Pt1 cells compared with parental IGROV‐1 cells (Figure 7A). Therefore, we further validated these findings in two cisplatin‐resistant OV cells, TUOS3 and OVISE^33^ (Figure S4). *SELENOI* knockdown significantly enhanced cisplatin's antiproliferative effects in a dose‐dependent manner, and increased intracellular ROS levels under cisplatin treatment (Figure 7B,C).

To identify potential targets for reversing platinum resistance by *SELENOI* silencing, we conducted an overlapping analysis of the identified 1164 DEGs with resistance‐related genes from the HESOC‐Platinum database (Figure 6K). A significant downregulation of Aldehyde Dehydrogenase 3 Family Member A1 (*ALDH3A1*), a key platinum resistance gene involved in ROS degradation,^37^ was observed in *SELENOI* knockdown cells (Figure 7D,E). A recent study demonstrated that the maintenance of high ALDH‐active cell subpopulations in tumors depends on Akt activation and is associated with functions including chemotherapy resistance and tumorigenicity.^38^ Interestingly, we found that *SELENOI* silencing reduced ALDH3A1 expression in parallel with decreased p‐Akt levels (Figure 7F). Conversely, *SELENOI* overexpression increased both Akt phosphorylation and ALDH3A1 protein levels (Figure 7G), enhancing cell proliferation and reducing cisplatin efficacy (Figure 7H,I). Treatment with Akt inhibitor MK2206 significantly restored cisplatin sensitivity and suppressed both p‐Akt and ALDH3A1 expression in *SELENOI*‐overexpressing cells (Figure 7J). Additionally, treatment with selective ALDH3A1 inhibitor CB7^39^, ^40^ reversed cisplatin resistance in these cells, leading to a significant decrease in cell viability (Figure 7K). Consistent with in vitro findings, *SELENOI* knockdown significantly reduced tumor growth in vivo (Figure 7L), and this effect was further enhanced by cisplatin treatment in the TUOS3–shSELENOI group (*p* < 0.05) (Figure 7M,N), with no observed adverse effects on mouse behavior, body weight, or organ pathology (Figures 7P and S5). These results indicate that *SELENOI* silencing could enhance cisplatin cytotoxicity in OV cells by impairing Akt phosphorylation and downregulating ALDH3A1.

### 2.8 Identification of celastrol as a potential compound targeting ovarian tumors with high SELENOI expression

**FIGURE 8:**
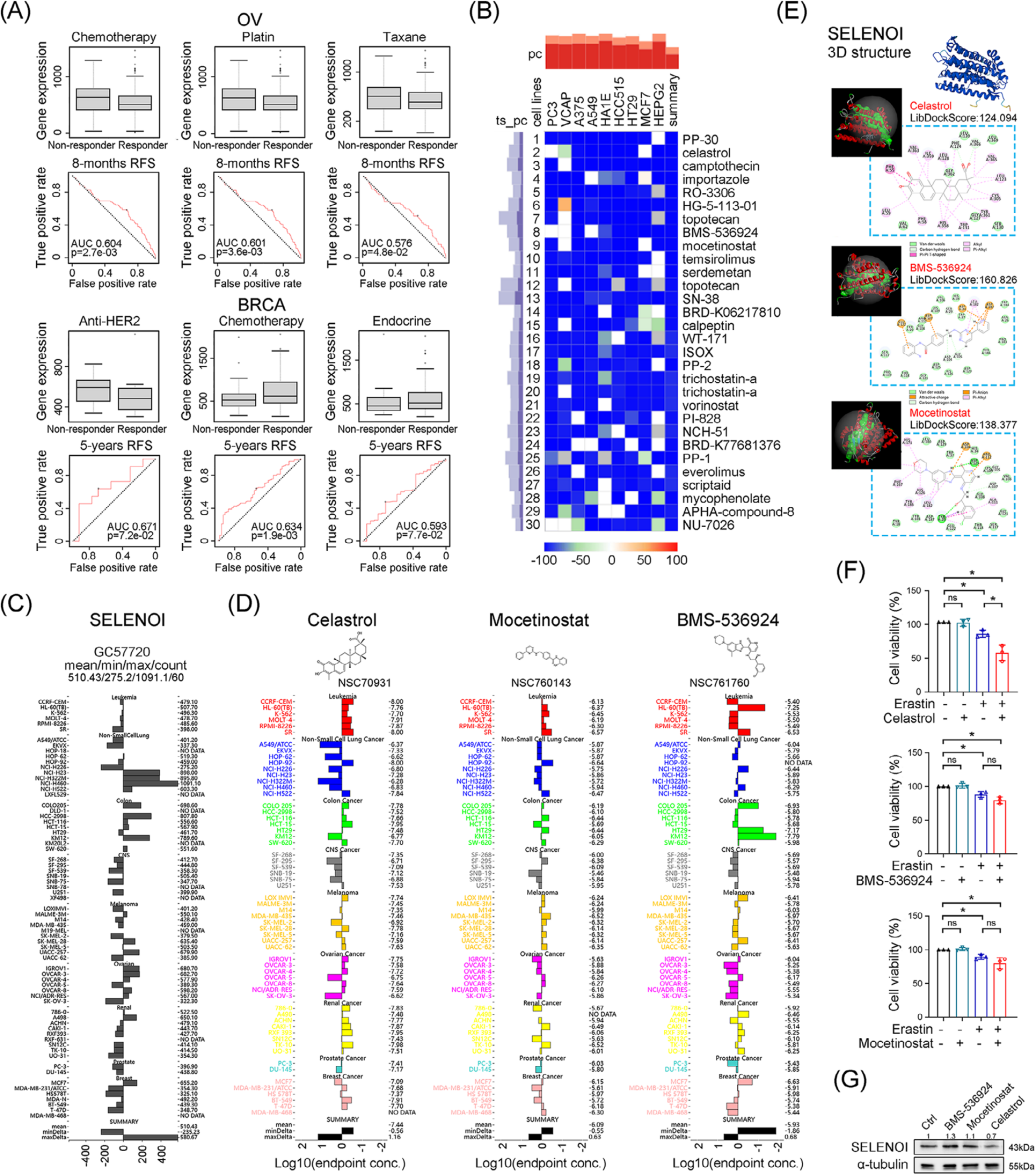
SELENOI predicted ovarian cancer treatment response and docked with SELENOI targeting compounds. (A) Analysis of SELENOI expression between chemotherapy responders and nonresponders in OV and BRCA patients and the ROC curve for predicting treatment accuracy using ROCplotter. (B) Heatmap showing the top 30 candidate SELENOI‐inhibiting compounds screened using cMap. Color bar and block colors represented similarity scores. (C and D) Comparison of GI50 (D) and SELENOI expression (C) of celastrol, BMS‐536924, and mocetinostat in cancer cell lines from the NCI60 project using COMPARE. The midline represented the mean log_10_ (GI50) or mean SELENOI expression. (E) 3D view showing the protein constructed through homology modeling, with the SELENOI drug interaction pocket shown in the upper left image. The 2D view within the dashed box showing the 2D structure of the drug, interacting amino residues, molecular forces, and molecular spatial distances. Visualization was conducted with Discovery Studio software. (F) Cell viability analysis of TUOS3 cells after treatment with erastin alone (10 µM), and in combination with celastrol (2.5 µM), mocetinostat (2.5 µM), or BMS‐536924 (2.5 µM) for 24 h. (G) Western blotting of SELENOI expression levels in TUOS3 cells treated with celastrol (2.5 µM), mocetinostat (2.5 µM), or BMS‐536924 (2.5 µM) for 24 h. α‐Tubulin was used as loading control. ^*^ *p* < 0.05, ^**^ *p* < 0.01, ^***^ *p* < 0.001; ns, not significant. Data represent means ± SD collected from three independent experiments.

To validate SELENOI's role in predicting treatment response, we investigated its correlation with treatment response in four cancers (BRCA, OV, GBM, and CRC) by using ROCplotter. In OV, high *SELENOI* expression was associated with poor chemotherapy response (Figure 8A), particularly in patients resistant to cisplatin and paclitaxel, the first‐line chemotherapy drugs. In BRCA, nonresponders to anti‐HER2 therapy exhibited higher *SELENOI* levels, while nonresponders to chemotherapy and endocrine therapy showed lower levels (Figure 8A). Given the reduced efficacy of platinum‐based chemotherapy in OV patients with high *SELENOI* expression, we sought more effective anti‐SELENOI drugs to improve the treatment outcomes. Using cMap, we identified 30 compounds that induce transcriptional changes opposite to those associated with high *SELENOI* expression (Figure 8B). We compared the GI50 values of the top 10 compounds across pan‐cancer cell lines using the COMPARE tool. Data for PP‐30, importazole, RO‐3306, and HG‐5‐113‐01 were unavailable. We then compared *SELENOI* expression in pan‐cancer cell lines with GI50 values for celastrol, BMS‐536924, and mocetinostat (Figure 8C,D). Celastrol exhibited the best fit with an average −log10 (GI50) of 7.44, and higher *SELENOI* expression in OV cell lines correlated with increased GI50 for celastrol (Figure 8D). Homology modeling and molecular docking using the SELENOI protein structure from alphaFold2.0 revealed that celastrol, BMS‐536924, and mocetinostat successfully docked with SELENOI, with the highest LibDockScores of 124.094, 160.826, and 138.377, respectively. The docking pocket structure and interaction details are shown in Figure 8E. Celastrol interacted with SELENOI residues through multiple types of interactions. Next, we performed in vitro experiments to assess these candidate small molecule inhibitors of SELENOI. Celastrol, BMS‐536924, and mocetinostat did not significantly inhibit the cell viability of OV cells at low doses individually (Figure 8F). However, combining low‐dose erastin with celastrol significantly reduced cell viability in TUOS3 cells, whereas other combinations showed no significant effect (Figure 8F). Additionally, SELENOI expression was decreased in TUOS3 cells treated with celastrol (Figure 8G). These findings suggest that celastrol could be a promising SELENOI‐targeting drug and a potential alternative therapy for platinum‐resistant OV.

## 3 DISCUSSION

The etiology of OV is multifactorial, involving genetic, dietary, and environmental factors. Key deletions or mutations in genes, including *TP53*, *BRCA*1/2, and *PTEN*, are closely linked to OV development and progression.^41^, ^42^ However, predicting OV progression and prognosis remains challenging due to the low penetrance and specificity of most discovered genes/proteins. The importance of selenium in endocrine‐related cancers, including breast cancer, prostate cancer, and OV,^43^, ^44^, ^45^ suggests that selenoproteins may serve as potential therapeutic targets.

SELENOI, a predominantly cytoplasmic selenoprotein, may function in the endoplasmic reticulum or Golgi apparatus, although its exact cellular localization remains controversial.^20^, ^46^, ^47^ Only recently has the physiological function of SELENOI in the de novo synthesis of PE has been elucidated, but evidence remains sparse. To date, the impact of SELENOI on human cancer is unclear. Our study reveals high expression of *SELENOI* in various malignancies. This expression pattern was confirmed in our OV cell line and patient cohort. Notably, elevated *SELENOI* levels were observed in high‐grade serous and advanced metastatic cancers, which are often resistant to platinum‐based chemotherapies and linked to poor outcomes.^7^, ^48^ Higher *SELENOI* was also associated with poor prognosis in three gynecological malignancies (OV, CESC, and UCEC), suggesting SELENOI as a potential biomarker. This is the first report of the carcinogenic role of SELENOI in a clinical pathology context. Ahmed et al. and Horibata et al. reported that *SELENOI* mutation was associated with severe complicated hereditary spastic paraplegia.^23^, ^24^ While *SELENOI* mutations are rare in tumors according to the TCGA database, its role in phospholipid synthesis, and plasma membrane integrity, highlights its potential importance in cancer biology.^49^, ^50^ Considering that *SELENOI* knockdown attenuates malignant phenotypes and promotes cell cycle arrest, these findings suggest a critical role of SELENOI in tumor progression and prognosis prediction.

Previous studies have not demonstrated SELENOI's role in cancer immunity. Our findings reveal a significant association between high *SELENOI* expression and reduced immune infiltration, immunosuppressive cancer subtypes, and poor antitumor immune responses across various solid tumors. Significant upregulation of *CD274* expression was observed in tumors with high *SELENOI* expression, including gynecological cancers. In OV and UCS, high *SELENOI* expression correlated with decreased abundance of stromal and immune cells, resulting in lower ESTIMATE scores. Elevated *SELENOI* levels were also prevalent in OV patients unresponsive to anti‐immunotherapy and anti‐PD‐L1 therapy, suggesting its potential as a predictive marker for antitumor immune response. Additionally, *SELENOI* was associated with CD8^+^ T cell suppression and immunotherapy resistance, indicating its potential as a target to enhance immunotherapy. We also verified a strong positive correlation between *SELENOI* expression and CD4^+^ T cell presence in most cancer types. CD4^+^ T helper (Th) cells are critical for initiating and sustaining anticancer immune responses and their differentiation relies on enzymes like SELENOI involved in the Kennedy pathway.^25^, ^26^ These findings suggest SELENOI could be a biomarker for CD4^+^ T cell infiltration. This study is the first to delineate a distinct association between SELENOI and cancer immunity, though further research and clinical validation are needed.

Previous studies have indicated that activated ACSL4 facilitates unsaturated fatty acids‐containing phospholipid biosynthesis, thus triggering lipid peroxidation and promoting ferroptosis.^12^, ^18^, ^19^ Consistently, our study demonstrates that knocking down *SELENOI* upregulates unsaturated fatty acid synthesis and impairs Akt phosphorylation, leading to ACSL4‐dependent ferroptosis. This also aligns with recent findings that activation of the PI3K/Akt pathway promotes resistance to oxidative stress and ferroptosis through SREBP1/SCD1‐driven lipogenesis.^32^ While an alternative pathway exists for PE synthesis in the inner mitochondrial membrane, SELENOI's role in the de novo biosynthesis of PE via the Kennedy pathway, particularly unsaturated plasmalogens, is indispensable.^20^, ^24^ Previous studies showed that plasmalogens protect hippocampal neurons by enhancing GPCR‐mediated Akt phosphorylation^51^ and maintain normal axonal integrity by regulating the Akt–ULK1 axis.^52^ We found that *SELENOI* silencing impairs Akt phosphorylation and its downstream effects, which is consistent with the effects of insufficient SELENOI‐dependent plasmalogen synthesis. Given the PI3K/Akt pathway's crucial role in tumor proliferation, invasion, and cell fate,^53^, ^54^ strategies to inhibit SELENOI levels could improve OV patient prognosis. Notably, tumor growth decreased in TUOS3–shSELENOI xenografts, providing direct evidence of SELENOI‐mediated tumor suppression.

Resistance to first‐line chemotherapy is a major challenge in treating advanced or recurrent OV.^7^ Previous studies have demonstrated that ALDH3A1, a member of the ALDH superfamily, enhances DNA damage repair and mediates chemoresistance through eliminating cellular ROS in cancer cells.^37^, ^55^, ^56^ Our transcriptomic analysis revealed that *SELENOI* knockdown significantly affected platinum resistance pathways, suggesting that inhibiting SELENOI may enhance sensitivity to chemotherapy. *SELENOI* knockdown increased OV cells’ sensitivity to cisplatin by inhibiting Akt phosphorylation and reducing ALDH3A1 expression. Moreover, treating *SELENOI*‐overexpressing cells with Akt inhibitor restored cell viability and sensitivity to cisplatin. This is also consistent with a recent study linking high ALDH activity and Akt activation to chemoresistance and tumorigenicity.^38^ In this study, *SELENOI* knockdown also resulted in elevated ROS levels. Increased ROS levels influence various signaling pathways, and thereby affecting cell survival and cisplatin sensitivity.^53^, ^54^ Since SELENOI is highly expressed in OV, inhibiting its function may offer a new approach to suppress tumor progression and improve OV chemosensitivity. However, further studies are needed to elucidate SELENOI's antitumor functions, and its underlying mechanisms, as well as potential side effects of targeting SELENOI to fully understand its impact in OV and other cancers.

In conclusion, our study demonstrated the ubiquitous upregulation of SELENOI in human malignancies and its role in ferroptosis, tumor immunity, and chemotherapy resistance. High *SELENOI* expression in female cancers, particularly OV, correlates with increased chemotherapy resistance, altered immunotherapy responses, and poor prognosis. Silencing *SELENOI* attenuated the malignant phenotype, reduced platinum drug resistance, and enhanced sensitivity to ferroptosis inducers in OV cells. Targeting SELENOI represents a promising therapeutic strategy for patients resistant to conventional treatments.

## 4 MATERIALS AND METHODS

### 4.1 Identification of significantly differential genes in OV

RNA‐seq data from 33 human cancers and matched normal samples were obtained from the TCGA and GTEx datasets. Four OV datasets (GSE18520, GSE27651, GSE14407, and GSE38666) and two uterine sarcoma datasets (GSE45510 and GSE36610) were collected from the GEO database. Differential gene expression was analyzed using R package limma.^57^ Genes with |log_2_foldchange| > 1 and *p* value < 0.05 were deemed statistically significant. GO and KEGG enrichment analyses of DEGs or coexpressed genes were performed using KOBAS‐i version 3.0.^58^

### 4.2 Differential expression, interaction network, functional enrichment, and prognosis analysis of SELENOI in pan‐cancer

Drug sensitivity data for SELENOI were sourced from the CTRP dataset.^59^ SELENOI protein expression in malignant and normal tissues was analyzed using the CPTAC database.^60^ Gene information within the GGI network was retrieved from the GeneMANIA,^61^ and protein data within the PPI network were collected from the STRING.^62^ Cytoscape version 3.9 with the Cytohubba plug‐in were employed to identify and visualize hub genes based on the betweenness centrality (BC) scores.^63^ Coexpressed genes with *SELENOI* across 33 cancers were identified using GEPIA2.0.^64^ Prognostic analysis of SELENOI in cancers was conducted using the PrognoScan^65^ and Kaplan–Meier plotter platform.^66^ Differential KEGG pathways between *SELENOI*‐high and ‐low groups were investigated using GSEA.^67^ Data analysis and visualization were carried out with GraphPad Prism 8.0 and bioinformatics platform (last accessed on June 20, 2024), an online platform for data analysis and visualization [http://www.bioinformatics.com.cn/].^68^ Figures and statistical analysis were automatically generated by the respective websites and platforms.

### 4.3 Correlation of SELENOI expression and immune cell infiltration, tumor microenvironment, and immunotherapy response in pan‐cancer

The EPIC algorithm was employed to assess the correlation between *SELENOI* expression and immune cell infiltration.^69^ Data from single cell dataset GSE181297 and tools for visualizing *SELENOI* expression were obtained from the CDCP.^70^ The R package maftools was employed to assess the correlation of *SELENOI* expression with TMB and MSI.^71^, ^72^ The R package ESTIMATE was utilized to assess *SELENOI* expression in the tumor tissue microenvironment.^73^ SELENOI's role in T cell dysfunction and CTL‐related prognosis across cancers was conducted using the TIDE.^74^ Relevance of SELENOI expression with treatment response as well as the ROC curves for treatment‐related survival were assessed using the ROCplotter.^75^

### 4.4 Correlation of SELENOI and ferroptosis, platinum resistance, as well as SELENOI‐targeting compounds screening and molecular docking analysis

Candidate genes regulated by SELENOI in ferroptosis were analyzed using the FerrDb.^76^ The HESOC‐Platinum database was used to examine SELENOI‐regulated candidate genes influencing cisplatin resistance.^77^ Anti‐SELENOI compounds were screened using the Connectivity Map (cMap),^78^ a heat map displaying the top 30 compounds was generated. SELENOI expression and GI50 concentrations for the top 10 cMap compounds were analyzed using the NCI COMPARE tool.^79^ Homology modeling of SELENOI proteins was performed using the AlphaFold database and AlphaFold2 software to study protein–compound interactions^80^ and the FASTA sequence listed in Table S4. Docking was conducted with Discovery Studio software 4.5 (Biovia, USA), using LibDock to analyze SELENOI–ligand interactions.

### 4.5 Reagents, antibodies, and cell lines

Cisplatin (S1166), MK2206 (S1078), SC79 (S7863), rosiglitazone (S2556), CB7 (F668527), puromycin (S7417), erastin (E7781), sorafenib (SML2653), celastrol (CM00992), BMS‐536924 (CM04766), mocetinostat (CM04416), DMSO (anhydrous, D12345), and propidium iodide (PI; P1304MP) were obtained from Selleck Chemicals (Houston, TX, USA), Proteintech (Wuhan, Hubei, China), Aladdin (Shanghai, China), Invitrogen and ThermoFisher Scientific (Waltham, MA, USA), and Sigma (St. Louis, MO, USA). Antibodies, including SELENOI (ab157571), Akt (9272S), Phospho‐Akt (Ser473) (4060S), p21 (DCS60), CDK4 (D9G3E), Cyclin D1 (92G2), ACSL4 (sc‐365230), ALDH3A1 (sc‐376089), α‐tubulin (sc‐5286), and GAPDH (D4C6R), were purchased from Cell Signaling Technology (Danvers, MA, USA) and Santa Cruz Biotechnology (Dallas, TX, USA). Lipofectamine 2000 was purchased from Invitrogen. All drugs were dissolved in DMSO per the manufacturer's guidelines and stored at −20°C. Cisplatin preparations were made fresh to prevent degradation.

Human ovarian immortalized cell line HOSE11‐12 and cancer cell lines (SKOV3, OVTOKO, OVCA420, TUOS3, OVISE, and KOC7C) were obtained from Procell (Wuhan, Hubei, China) and MeisenCTCC (Hangzhou, Zhejiang, China). OVTOKO, TUOS3, OVISE, and KOC7C were cultured in DMEM/F12 medium (Invitrogen), whereas SKOV3 and OVCA420 cells were cultured in M199:M105 (Sigma–Aldrich) and RPMI 1640 (Gibco), respectively. HOSE11‐12 cells were cultured in DMEM (Invitrogen). All medium was supplemented with 10% fetal bovine serum (Gibco) and 1% penicillin–streptomycin (Gibco). Cell lines were maintained at 37°C in 5% CO_2_ atmosphere. All cell lines used were STR‐identified and mycoplasma contamination was routinely monitored using LookOut PCR detection kit (Sigma).

### 4.6 Lentivirus‐mediated knockdown and overexpression of SELENOI

Lentiviral plasmids (pLV2–U6–*SELENOI* (human)–shRNA–EGFP–puro, pLV2–U6–EGFP–puro vector, pCDH–CMV–*SELENOI*–EF1a–copGFP, pCDH–EF1a–copGFP vector) and 2nd packaging system (psPAX2 and pMD2.G) were purchased from Miaoling Plasmid Platform (Wuhan, Hubei, China). To construct SELENOI‐targeting lentiviral shRNA plasmids, commercially validated oligonucleotides targeting *SELENOI* (shSELENOI‐1, 5′‐CGGCACTAACTCATGGTT CTA‐3′, TRC clone ID: TRCN0000158136; shSELENOI‐2, 5′‐GCTACATCCTAGAGTATTCTA‐3′, TRC clone ID: TRCN0000151614; Sigma–Aldrich) were inserted into the pLV2‐EGFP empty vector (Addgene). Lentiviral particles were generated by cotransfecting 293T cells with *SELENOI*‐targeting shRNA plasmids or overexpression plasmids, along with psPAX2 and pMD2.G. Infected cells were cultured in medium containing 5 µg/mL puromycin for 2 weeks to establish stable cell lines. Among the tested shRNAs, shSELENOI‐2 was selected for further experiments due to its superior knockdown efficiency (Figure S1).

### 4.7 Western blotting

Protein extracts were prepared using a RIPA‐based extraction kit (ThermoFisher Scientific). Proteins were separated using 6–12% SDS‐PAGE (Invitrogen), then transferred onto Immun‐Blot PVDF membranes (Bio‐Rad Laboratories, Hercules, USA). Membranes were blocked for 1 h at room temperature, then incubated overnight at 4°C with primary antibodies, followed by 1 h with secondary antibodies. After washing with 1×TBS‐T, membranes were incubated with WesternBright ECL HRP substrate (Advansta, Menlo Park, USA), and detection was conducted using an Amersham Imager 680 (GE Healthcare, Chicago, USA).

### 4.8 RT quantitative PCR

Total RNA was extracted using Trizol (Invitrogen) and reverse transcribed with PrimeScript RT Master Mix (Takara, Mountain View, USA). qPCR was performed on a LightCycler 480 (Roche, Basel, Switzerland) using HotStart SYBR Green qPCR Master Mix (ExCell Bio, Shanghai, China). Relative mRNA levels were quantified using the ΔΔCt method.^81^ Validated qPCR primers are available upon request.

### 4.9 RNA‐sequencing

RNA isolated from TUOS3–shNC and TUOS3–shSELENOI cells was submitted to BGI Technology Services Co., Ltd. (Shenzhen, Guangdong, China) for high‐throughput sequencing and subsequent bioinformatics analysis. Library construction and sequencing were carried out on the BGISEQ‐500 platform. Quality assessment of the sequencing reads was conducted using FastQC and MultiQC, followed by trimming of adapters and low‐quality bases using Trimmomatic to ensure clean reads for analysis. DEGs were identified using DESeq2, with a threshold of |log_2_foldchange| > 1 and *p* value < 0.05. KEGG pathway enrichment analysis of DEGs was carried out with KOBAS‐i.

### 4.10 Cell viability, colony formation, and 3D sphere formation assays

For cell viability assays, 1000–5000 cells were plated in 96‐well plates. MTT solution (1 mg/mL; Sigma–Aldrich) was added per well and plates were incubated at 37°C for 3 h. Formazan was dissolved with DMSO, and absorbance at 595 nm was measured using a Tecan microplate reader (Tecan, Grodig, Austria). For clonogenic assays, 1000 cells were plated in six‐well plates and incubated for 10 days at 37°C. Then, colonies were fixed, stained with 0.1% crystal violet (Sigma–Aldrich) in 50% methanol, and imaged using an Amersham Imager 680. Colony areas were quantified using ImageJ with the ColonyArea plugin. For 3D spheroidization assays, 1000 cells were seeded in Nunclon Sphera 96‐well ultra‐low attachment plates (ThermoFisher) and cultured for 7 days. Spheroids were imaged for size and morphology using a Nikon imaging system.

### 4.11 Cell migration and invasion assays

For cell migration assays, 1 × 10^5^ cells were plated in serum‐free Boyden chambers (Corning Life Sciences, Tewksbury, MA). For invasion assays, cells were plated in chambers precoated with Matrigel (Corning). Chambers were placed in 24‐well plates with 10% FBS DMEM/F12 medium to create a chemoattractant gradient. After 24 h, migrated or invaded cells were fixed with 4% paraformaldehyde, stained with 0.1% crystal violet and imaged with a Nikon Eclipse Ti2‐E imaging system (Nikon, Tokyo, Japan). The purple precipitate was extracted with ethanol, and absorbance at 570 nm was measured using a Tecan microplate reader.

### 4.12 Cell cycle and ROS analysis by flow cytometry

For cell cycle analysis, cells were plated in six‐well plates, incubated for 48 h, then trypsinized, centrifuged, and fixed in 70% ethanol overnight at 4°C. Cells were resuspended in 1×PBS with 20 µg/mL PI and 200 µg/mL RNaseA, incubated at 37°C for 30 min prior to analysis. For ROS analysis, cells were plated in six‐well plates, treated according to the experimental design, stained with 5 µM CellROX Green reagent for 30 min at 37°C. Poststaining, cells were trypsinized, centrifuged, and resuspended in 1×PBS, and kept on ice prior to analysis. Flow cytometric analysis was performed with a FACS LSRFortessa flow cytometer (BD Biosciences), with specific settings adjusted for PI or CellROX Green fluorescence.

### 4.13 GSH, MDA, and unsaturated fatty acids level measurement

GSH levels were assessed using the GSH‐Glo™ Glutathione kit (#V6911; Promega, Madison, USA). After drug treatment in 96‐well plates, 100 µL of GSH‐Glo™ reagent was added per well, incubated for 30 min, followed by 100 µL of luciferin detection reagent for 15 min. Luminescence was measured with a Tecan plate reader. MDA levels were measured using a Lipid Peroxidation Assay Kit (#ab118970; Abcam, MA, USA). After drug treatment, 300 µL of Developer VII/TBA reagent was added, incubated at 95°C for 60 min. After cooling, 100 µL of the reaction mixture was transferred to a new 96‐well plate, and absorbance at 532 nm was measured using a Tecan microplate reader. Total unsaturated fatty acid levels were measured using lipid extraction kit (#ab211044; Abcam) and lipid assay kit for unsaturated fatty acids (#ab242305; Abcam). Cells were plated in six‐well plates, incubated for 48 h, then trypsinized, centrifuged, and resuspended in 25 µL 1×PBS with 500 µL extraction buffer. The mixture was agitated for 15 min at room temperature, centrifuged, and the supernatant was collected and dried overnight at 37°C. The dried lipid extract was resuspended in 50 µL of suspension buffer and sonicated for 15 min at 37°C before quantification. Absorbance was measured at 540 nm.

### 4.14 Xenograft tumor model

Animal study was approved by the Institutional Animal Care and Use Committee of Shenzhen Second People's Hospital, the First Affiliated Hospital of Shenzhen University. Four‐week‐old female BALB/cAnN‐nu (Nude) mice were used for establishing the subcutaneous xenografts by injection of 1 × 10^8^ *SELEONI* knockdown or control TUOS3 cells into the flank of each mouse. After 30 days, mice were randomized into four groups: vehicle control, vehicle cisplatin, *SELENOI* knockdown, and SELENOI knockdown cisplatin. For drug treatment, saline vehicle (control) or cisplatin (5 mg/kg) were administered via intraperitoneal injections twice weekly for five doses. Tumor size and body weight were monitored, with tumor volume calculated as volume (mm^3^) = length × width^2^/2.^82^

### 4.15 Statistical analysis

Statistical analyses were conducted using IBM SPSS statistics version 25 (SPSS Inc, Chicago, USA). Differences in Western blotting, qPCR, xenograft mouse models, and functional assays (MTT, ROS, GSH, and MDA) were evaluated using Student *t*‐test, Mann–Whitney, or Kruskal–Wallis test as appropriate. Spearman correlation was used to assess the association between SELENOI and cancer prognosis, tumor immunity, ferroptosis, and platinum resistance. Data are presented as mean ± SD from at least three independent experiments. Statistical details and significance are also provided in figure notes. Two‐sides *p* values less than 0.05 were defined as significant.

## AUTHOR CONTRIBUTIONS

*Conception and design, methodology, experiment and data curation, supervision, writing—review and editing*: Jing Li *Data acquisition and analysis, writing—review and editing*: Mimi Chen *Data acquisition and analysis, writing—review and editing*: Dingwen Huang *Data acquisition and analysis, writing—review and editing*: Ziyin Li *Statistical analysis and biostatistics*: Y. C. *Statistical analysis and biostatistics*: Jinhua Huang *Clinical samples and data collection, writing—review and editing*: Mimi Chen *Western blotting, writing—review and editing*: Zhili Zhou *Conception and design, methodology, data curation, writing—review and editing*: Zhiying Yu All authors read and approved the final manuscript.

## CONFLICT OF INTEREST STATEMENT

The authors declare that they have no conflict of interest.

## ETHICS STATEMENT

The procedures involving human subjects in this study were approved by the Ethics Committee of the First Affiliated Hospital of Shenzhen University (Shenzhen Second People's Hospital) (2023‐069‐01‐YJ). Information about ovarian cancer tissue samples used in this study was obtained with the consent of the individuals for publication. All animal experimental procedures were also approved by the Committee on the Ethics of Animal Experiments of the First Affiliated Hospital of Shenzhen University (20220081).

## Supporting information



Supporting Information

Supporting Information

## Data Availability

Raw RNA sequencing data for shNC and shSELENOI experiments (related to Figure 6) are deposited in the National Genomics Data Center (NGDC) under accession number CRA016828. The processed data matrix is provided in Supporting Information tables. Links to publicly available datasets are included in the *Methods* section, along with descriptions of data analysis procedures. Additional data used or analyzed during this study are available from the corresponding author upon reasonable request.
