# Supplementary material for: Inhibition of Selenoprotein I promotes ferroptosis and reverses resistance to platinum chemotherapy by impairing Akt phosphorylation in ovarian cancer
Source: MedComm (2020). 2024 Dec 11;5(12):e70033. doi: 10.1002/mco2.70033 (PMC11635127; doi:10.1002/mco2.70033)
Supplement: Supplementary file 1 — Supporting Information [file MCO2-5-e70033-s002.docx]

**Title page：**

**Type of study: Original Research Article**

**Inhibition of Selenoprotein I promotes ferroptosis and reverses resistance to platinum chemotherapy by impairing Akt phosphorylation in ovarian cancer**

**Short Running title: Targeting SELENOI in ovarian cancer therapy**

**Jing Li^a,b, 1, ⁎^, Mimi Chen^a,1^, Dingwen Huang^a,1^, Ziyin Li^a,1^, Yu Chen^a^, Jinhua Huang^a^, Yuanqun Chen^a^, Zhili Zhou^c^, Zhiying Yu^a, ⁎^**

^a^ Department of Gynecology, Shenzhen Second People's Hospital, the First Affiliated Hospital of Shenzhen University, Shenzhen 518000, China.

^b^ Guangdong Key Laboratory for Biomedical Measurements and Ultrasound Imaging, National-Regional Key Technology Engineering Laboratory for Medical Ultrasound, School of Biomedical Engineering, Shenzhen University Medical School, Shenzhen 518060, China.

^c^ Department of Endocrinology and Metabolism, Nanfang Hospital, Southern Medical University, Guangzhou 510515, China.

^1^ These authors contributed equally to this work

^*^ **Corresponding author** at: 3002 Sungangxi Road, Futian District, Shenzhen, Guangdong, 518000, China.

E-mail address: [lizheyzy@163.com](mailto:lizheyzy@163.com) (ZY. Yu), [kriklee@connect.hku.hk](mailto:kriklee@connect.hku.hk) (J. Li).

Telephone number: 0086 13143469517


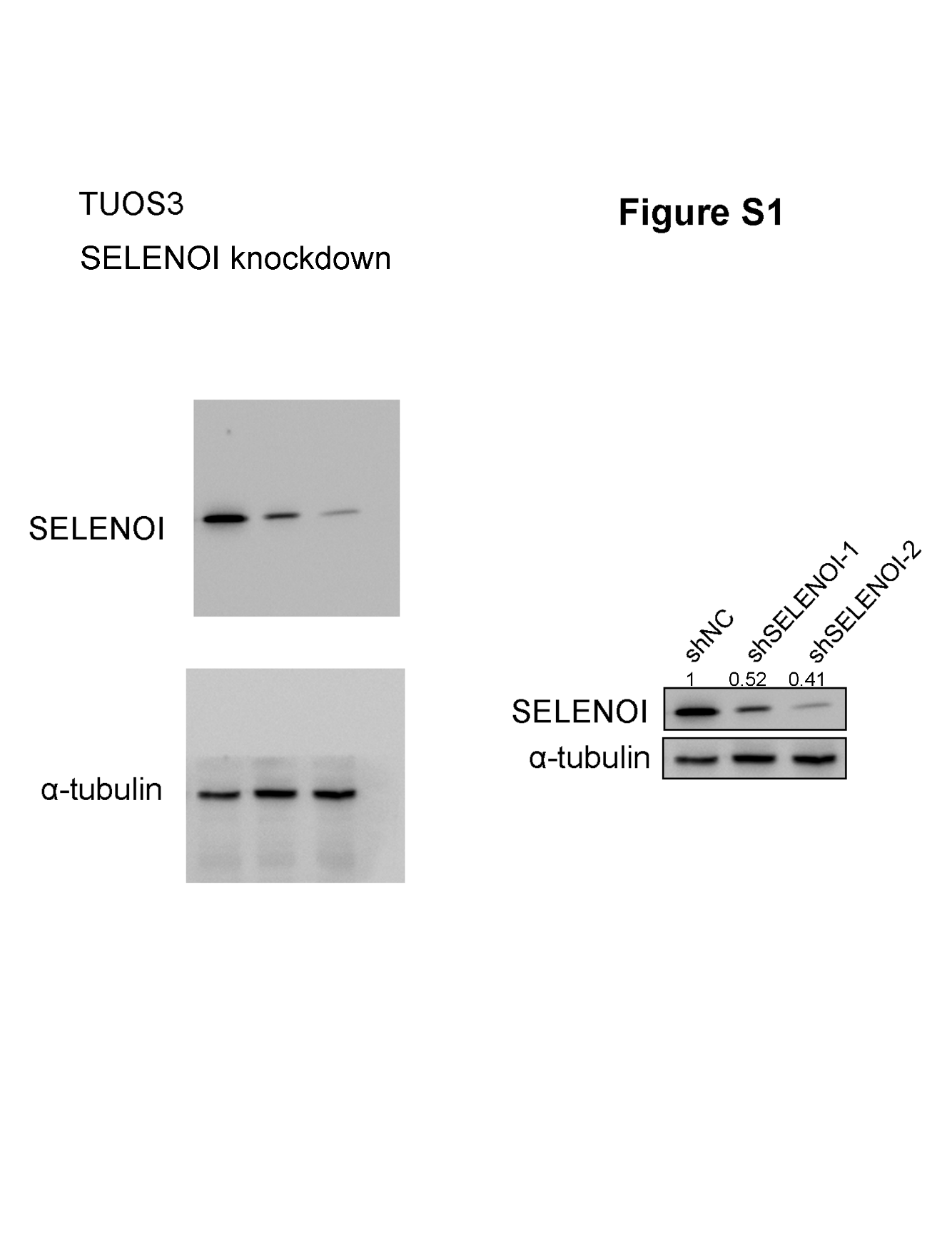


**FIGURE S1.** Western blotting analysis of SELENOI protein levels in *SELENOI* knockdown (shSELENOI-1 and shSELENOI-2) OV cells TUOS3. α-tubulin was used as loading control.


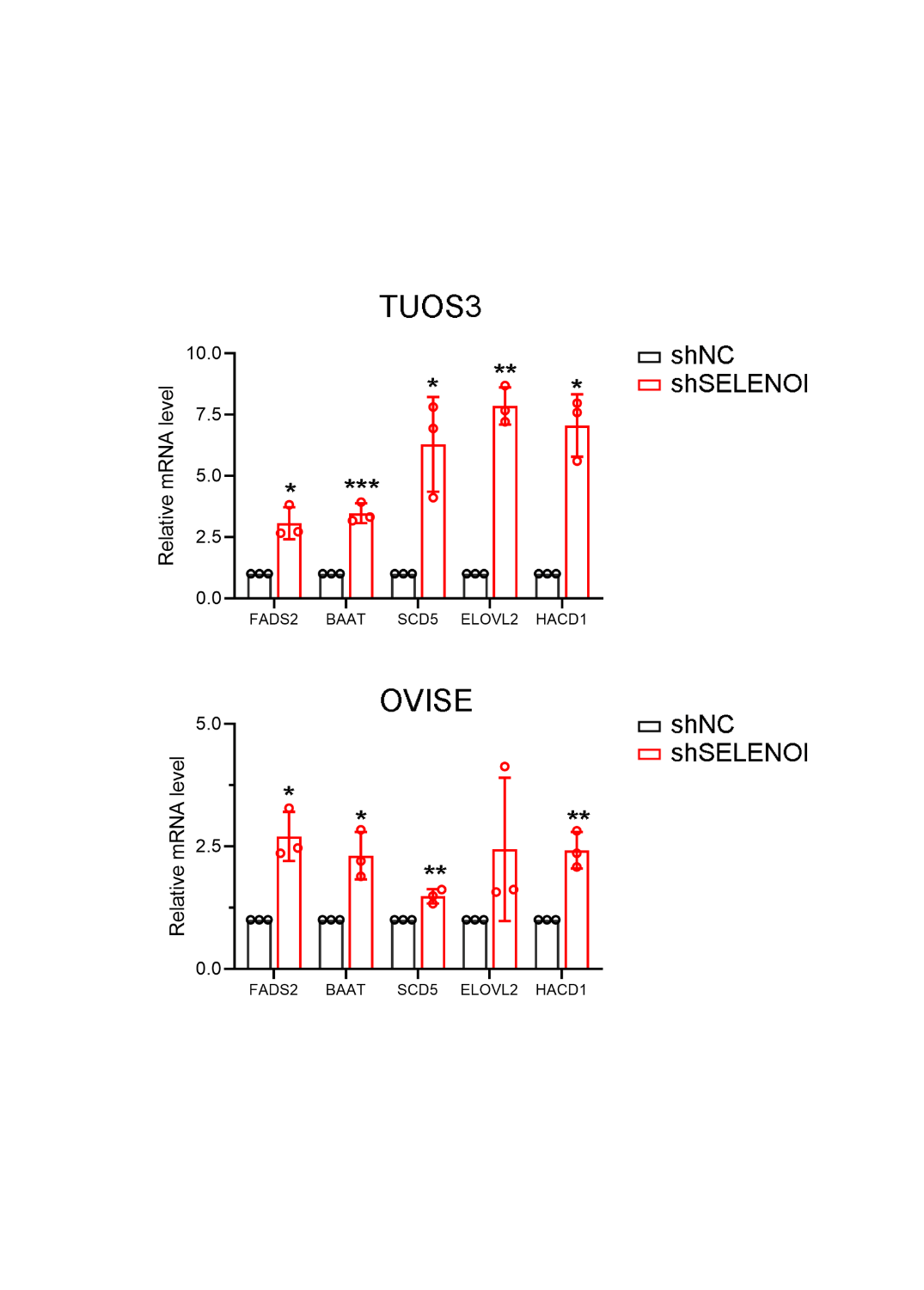


**FIGURE S2.** qPCR analysis of multiple key genes involved in unsaturated fatty acids synthesis pathway in *SELENOI* knockdown (shSELENOI) and control (shNC) OV cells. ^*^p < 0.05, ^**^p < 0.01, ^***^p < 0.001. Data represents means ± SD collected from three independent experiments.


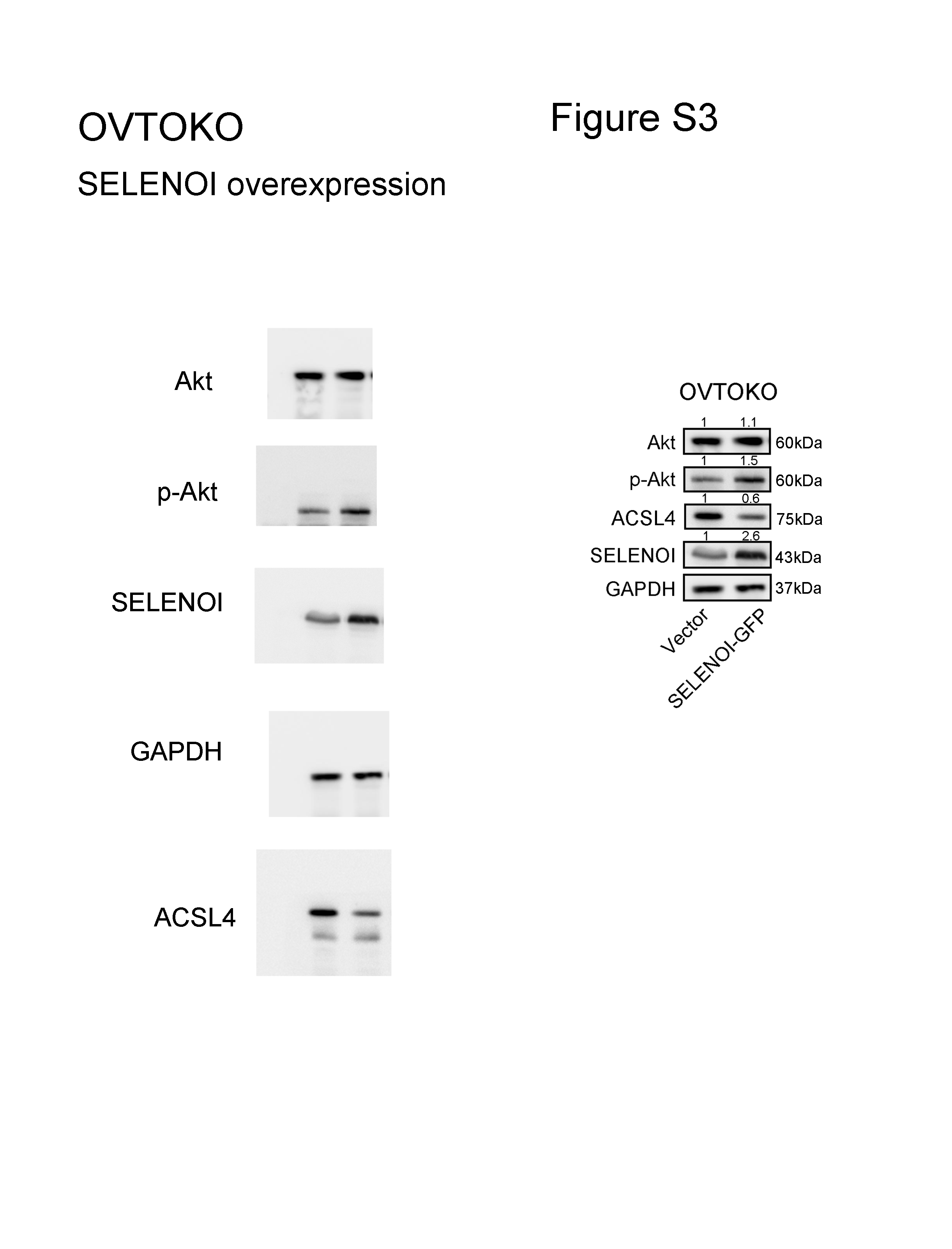


**FIGURE S3.** Western blotting analysis of Akt, p-Akt and ACSL4 expression levels in *SELENOI* overexpression OVTOKO and control cells. GAPDH was used as loading control.


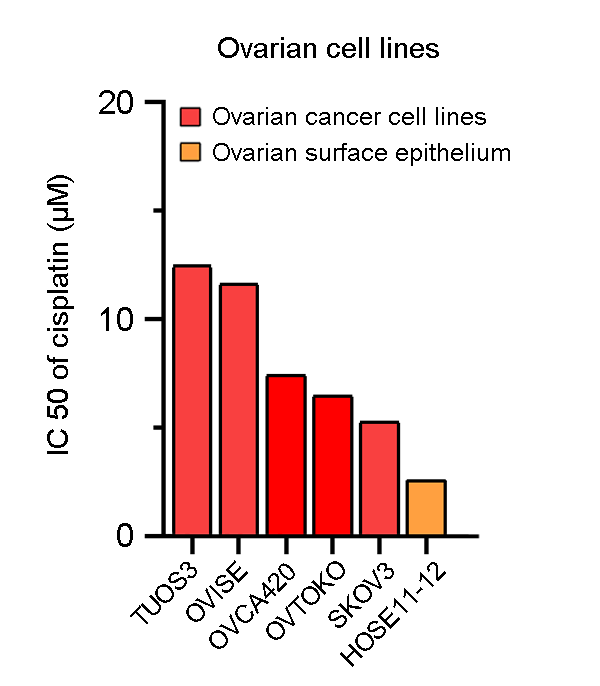


**FIGURE S4.** IC50 values of cisplatin in a cohort of ovarian cell lines. Data represents means ± SD collected from three independent experiments.


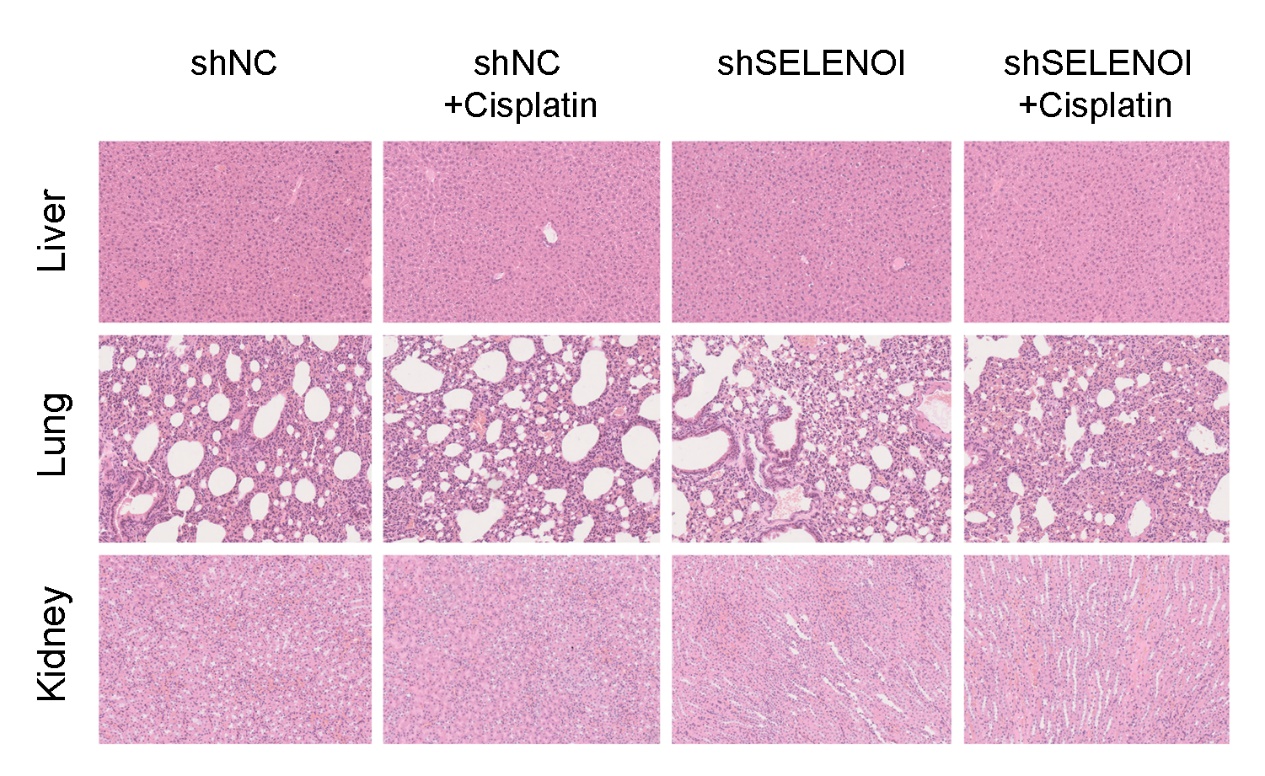


**FIGURE S5.** Immunohistochemical staining (IHC) analysis on the liver, lung, and kidney of the four groups of mice after cisplatin treatment.
